# Acute, sublethal, and combination effects of azadirachtin and *Bacillus thuringiensis* on the cotton bollworm, *Helicoverpa armigera*

**DOI:** 10.1093/jis/14.1.30

**Published:** 2014-01-01

**Authors:** Zahra Abedi, Moosa Saber, Samad Vojoudi, Vahid Mahdavi, Ehsan Parsaeyan

**Affiliations:** Department of Plant Protection, College of Agriculture, University of Maragheh, Maragheh, 55181-83111, Iran

**Keywords:** bioassay, biorational insecticides chemical control, life table

## Abstract

The cotton bollworm, *Helicoverpa armigera* Hübner (Lepidoptera: Noctuidae) is a polyphagous and cosmopolitan insect pest that causes damage to various plants. In this study, the lethal and sublethal effects of azadirachtin and *Bacillus thuringiensis* Berliner sub sp *. kurstaki* (Bacillales: Bacillaceae) were evaluated on third instar *H. armigera* under laboratory conditions. The LC50 values of azadirachtin and *Bt* were 12.95 and 96.8 µg a.i./mL, respectively. A total mortality of 56.7% was caused on third instar larvae when LC20 values of the insecticides were applied in combination with each other. The LT50 values of azadirachtin and *Bt* were 4.8 and 3.6 days, respectively. The results of the sublethal study showed that the application of LC30 value of azadirachtin and *Bt* reduced the larval and pupal weight and increased larval and pupal duration of *H. armigera* . The longevity and fecundity of female adults were affected significantly by the insecticides. Female fecundity was reduced by the treatments, respectively. The lowest adult emergence ratio and pupation ratio were observed in the azadirachtin treatment. The results indicated that both insecticides have high potential for controlling of the pest.

## Introduction

*Helicoverpa armigera* Hübner (Lepidoptera: Noctuidae) is one of the most destructive pests of field crops worldwide. It is a highly polyphagous, multivoltine, and economically-important pest of cotton and other crops and has developed resistance against most of the modern classes of synthetic insecticides (Forrester et al. 1993). Azadirachtin, a tetranortriterpenoid compound derived from the neem tree, *Azadirachta indica* A. Juss (Sapindales: Meliaceae), has insecticidal activity against phytophagous insects ( Spollen and Isman 1996 ). This active compound has several biological properties, including antifeedant effects ( Zehnder and Warthen 1988 ; Schmutterer 1990 ), insect growth regulator characteristics ( Ilio et al. 1999 ), and repellency ( Schmutterer 1990 ).

Neem pesticide has been effectively used against >400 species of insects, including many key crop pests, and has proven to be one of the most promising plant ingredients for integrated pest management ( Sahak et al. 2010 ). Neem extracts are usually safe for beneficial organisms, such as bees, predators and parasitoids, mammals, and also for the environment, with minimal residual effects ( Pavela 2009 ).

To prevent the damage that larvae produce in crops, a variety of methods are used for their control, including the use of chemical pesticides and microorganisms ( Avilla et al. 2005 ). The most important case of the latter is *Bacillus thuringiensis* Berliner (Bacillales: Bacillaceae), a bacterium that produces different proteins (δ-endotoxins) toxic to larvae of different species of Lepidoptera and other insects ( Schnepf et al. 1998 ). *Bt* has been used by spraying its spores and crystals on the pal-nts ( Avilla et al. 2005 ). The toxicity of *Bt* subspecies *kurstaki* and *aizawai* varies significantly among Lepidopteran species and life stages ( Mashtoly et al. 2011 ). Several authors have studied the effect of *Bt* toxins on *H. armigera* populations from China, India, and Australia ( Liao et al. 2002 ; Fengxia et al. 2004 ; Jalali et al. 2004 ). It has been shown in lepidoterous insects that the spores potentiated and synergized the insecticidal activity of the crystal protein ( Dubois and Dean 1995 ). Neem products can be mixed with other biopesticides, microbials, or with synergists ( Koppenhofer and Kaya 2000 ). Their favorable ecotoxicological profile and short period of persistence in the environment make these compounds a good choice for integrated pest management programs in vegetable crops ( Pineda et al. 2006 ). Sublethal effects may be manifested as reductions in life span, development rates, fecundity, changes in sex ratio, and changes in behavior ( Stark and Banks 2003 ).

The purpose of this study was to assess the lethal, sublethal, and combination effects of azadirachtin and *Bt* on *H. armigera* under laboratory conditions.

## Materials and Methods

### Insect culture

*H. armigera* larvae were collected from cotton fields in Moghan District of Ardebil Province, Iran, in 2011, and reared on an artificial diet ( Shorey and Hale 1965 ). For preventing can-nibalism, the third instar larvae were transferred into individual glass vials (3 ×9 cm) and were maintained until pupation. After adult appearance, 20 pairs of adult moths were placed into 20 ×30 cm plastic containers with a 1:1 sex ratio for mating and egg-laying. The adults were fed a 10% honey solution. *H. armigera* were reared at 26 ± 1°C, 70 ± 5% RH, and a photoperiod of 16:8 L:D under laboratory conditions.

### Insecticides

The insecticides used in the experiments were azadirachtin (Bioneem 0.09% EC, SaferBrand, www.saferbrand.com ) and *Bacillus thuringiensis* subsp. *kurstaki* strain ABTS-351 Solid (12.74% EC, SaferBrand).

### Bioassays

Newly-molted *H. armigera* third instar larvae were used for bioassay experiments, and were exposed to azadirachtin and *Bt* insecticides orally. The preliminary dose-setting experiments were carried out to determine the main concentrations of the bioassay test. The main concentrations were 25.2, 19.6, 15.2, 11.8, 9.2, and 7.2 µg a.i./mL for azadirachtin, and 229.3, 169.4, 125.1, 92.4, 66.9, and 51 µg a.i./mL for *Bt.* Then, 1 mL from each concentration was compounded into 9 mL of the artificial diet. After incorporation of the insecticides into the diet, 15 third instar larvae were transferred on the treated diet in the individual glass vials. Distilled water was used for the control group. Then, the glass vials were transferred to the growth chamber under the above-mentioned conditions. Mortality was recorded at intervals of 24 hr for seven days. Each concentration had three replications, and each experiment was replicated three times. The results of each trial were tested for lack of fit by using PROC GENMOD procedures ( Robertson et al. 2007 ; SAS Institute 2002 ), and the data were analyzed using PROC PROBIT ( SAS Institute 2002 ) to compute LC50 and LT50 values on a standard and log scale with associated 95% fiducial limits.

### Interaction effects

In this experiment, the LC20s of the insecticides alone were intitially assessed on third instars of *H. armigera* , then LC20s of both insecticides were mixed together and mortality was recorded for seven days for both experiments.

### Sublethal effects

The sublethal effects associated with azadirachtin and *Bt* were evaluated by using about 100 third instar *H. armigera* treated with LC30 of either insecticide. Larvae were allowed to feed on the treated diet in an individual glass vial for seven days, because the LC30s of these insecticides were calculated in the mentioned period of time. After seven days, the survivors were weighed and then kept in individual glass vials, where they fed on untreated artificial diet until pupation. The pupal weight and life span of pupae were recorded after pupation.

The influence of insecticides on fecundity and longevity was assessed by pairing moths in a small mating chamber lined and covered with chiffon. The mating chambers were provided with a 10% honey solution on a moist cotton trough that was replaced every day. The number of eggs laid by females was recorded daily until each female died. The data were analyzed by ANOVA with mean separation at a 5% level of significance by the LSD test.

## Results and Discussion

### Larval toxicity bioassay

Third instars of *H. armigera* were susceptible to azadirachtin and *Bt* incorporated into the diet. The LC50 values indicated that the toxicity of azadirachtin (12.95 µg a.i./mL) was higher than that of *Bt* (96.8 µg a.i./mL) ( Table 1 ). The results of LT50 studies of the insecticides are shown in Table 2 . These results showed that the effects of *Bt* were exhibited faster than azadirachtin. The LT50 values of azadirachtin and *Bt* did not differ significantly, because the fiducial limits did not overlap.

**Figure 1.**
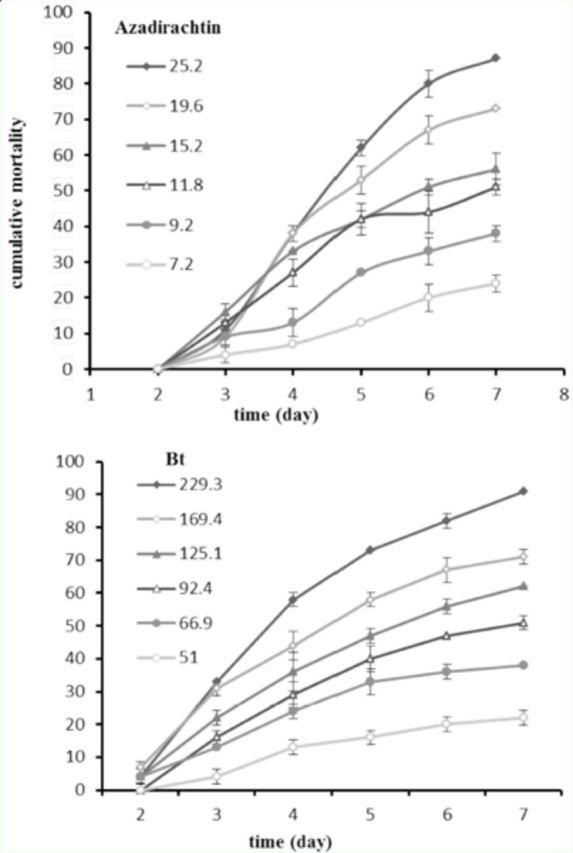
Cumulative percentage mortality (corrected ± SE) on third instar larvae of *Helicoverpa armigera* after exposure to different concentrations (μg a.i./mL) of azadirachtin and *Bt.* High quality figures are available online.

The cumulative percentage mortality on third instar larvae of *H. armigera* after exposure to different concentrations of azadirachtin and *Bt* for 7 days is shown in Figure 1 .

**Table 1.**
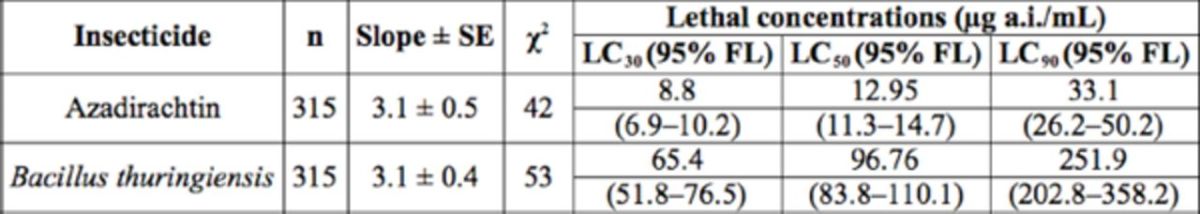
Toxicity of azadirachtin and *Bt* on third instar larvae of *Helicoverpa armigera.*

Lethal concentrations and 95% fiducial limits (FL) were estimated using logistic regression.

**Table 2.**
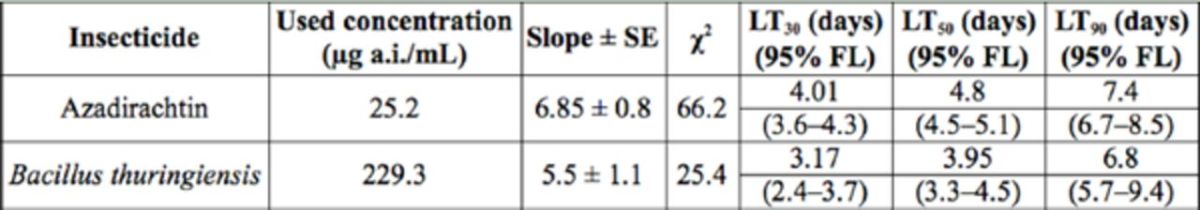
LT50 values of azadirachtin and *Bt* on third instar larvae of *Helicoverpa armigera.*

Lethal times and 95% fiducial limits (FL) were estimated using logistic regression.

The results showed that both insecticides had toxic effects on third instar larvae of *H. armigera* , although the toxicity of azadirachtin was higher than that of *Bt* . Izadyar et al. (2005) reported that the LC50 and LT50 values of *Bt* (DiPel) were 8 ×10 ^6^ CFU/mL and 3.8 days on *H. armigera* , respectively.

Rao et al. (1995) showed that the LC50 values for neonate and the second instar larvae of *H. armigera* were 0.002 and 0.004 % when fed NeemAzal-treated cotton leaves continuously. The LC50 values were 0.005, 0.02, and 0.03% for the first, second, and third instar larvae of *H. armigera* when the exposure was limited to 48 hr. Furthermore, they reported that the concentration of 200 ppm of NeemAzal significantly reduced larval and pupal weight in comparison with control.

### Sublethal effects

Larval exposure to an LC30 of the insecticides resulted in a significant reduction in pupal (F = 80.9; df = 2, 175; *P* & 0.0001) and larval weight (F = 104.3; df = 2, 245; *P* & 0.0001) compared to the control. Significant extensions in the durations of the larval (F = 253.9; df = 2, 191; *P* & 0.0001) and pupal stages (F = 65.5; df = 2, 158; *P* & 0.0001) were observed in the treatments compared with the control ( Table 3 ). The sublethal effects of insecticides on longevity and fecundity of female *H. armigera* are shown in Table 3 . The longevity of female adults was affected significantly by the insecticides (F = 7.9; df = 2, 37; *P* = 0.0015), and the control had higher longevity compared to the treatments. Longevity was reduced by 18.1% and 29.4% by azadirachtin and *Bt* treatments, respectively, compared to the control. The mean number of eggs per female (Mx) (F = 0.7; df = 2, 37; *P* = 0.0002) was affected by the insecticides ( Table 3 ). Female fecundity was reduced by 29.2% and 18.4 % by azadirachtin and *Bt,* respectively. Both insecticides had a significant effect on the oviposition of *H. armigera* adults.

The LC30 was chosen as a low lethal concentration for sublethal effects studies because it is the mortality threshold (30%) recommended for the use of pesticides in integrated pest management ( Desneux et al. 2007 ), and therefore it is crucial in assessing possible sublethal effects on pests. These sublethal effects should be evaluated because they could have a strong impact on the population dynamics of this lepidopteran pest and could contribute to its management ( Pineda et al. 2009 ). In this study, some of the biological parameters, such as longevity, fecundity, pupal formation, and adult emergence, of *H. armigera* were evaluated after exposure to azadirachtin and *Bt* .

**Table 3.**
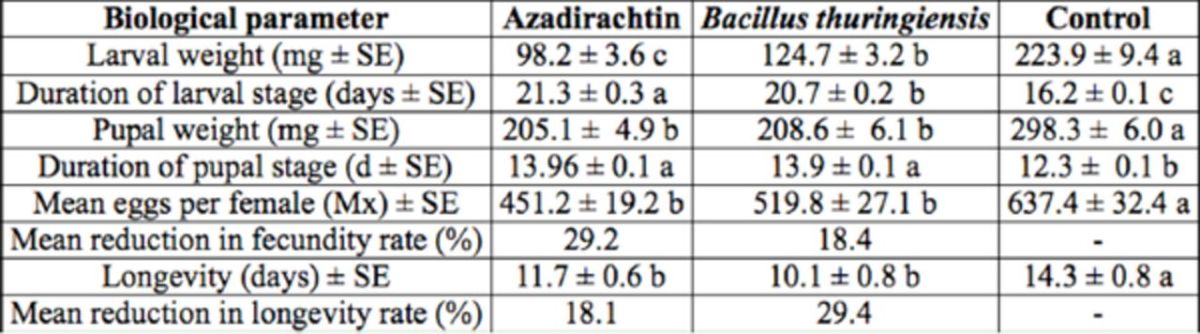
Sublethal effects of LC30 values of azadirachtin (8.8 μg a.i./mL) and *Bt* (65.4 μg a.i./mL) on biological parameters of *Helicoverpa armigera.*

Means within a row followed by different letters are significantly different (Fisher’s protected least significant difference; *P* & 0.05)

Heravi et al. (2009) studied the antifeedant, growth deterrent, and repellency characteristics of formulations of azadirachtin such as NeemAzal and NeemPlus on third instar larvae of *H. armigera.* In their study, all parameters were significantly affected by treatments, and none of the larvae reached pupal stage. LT50 values were 4.13 days and 7.68 days for NeemAzal and NeemPlus, respectively.

Ma et al. (2000) studied the toxicity and biological effects of azadirachtin on first and second instar larvae of *H. armigera.* High mortality of larvae, growth retardation, including reduced larval and pupal weight, and extension of development were observed in the treatment. Similar effects were observed in our study. In another study, azadirachtin reduced the adult longevity of *Spodoptera littoralis* when it was applied orally ( Pineda et al 2009 ).

**Figure 2.**
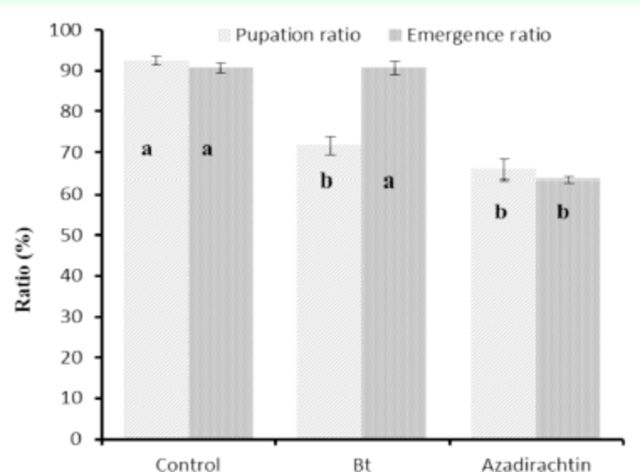
Post-exposure effects of azadirachtin and *Bt* on the pupation and adult emergence of *Helicoverpa armigera.* Data marked with different letters differ significantly ( *P* ≤ 0.05) based on the least significant difference multiple comparison test. High quality figures are available online.

**Figure 3.**
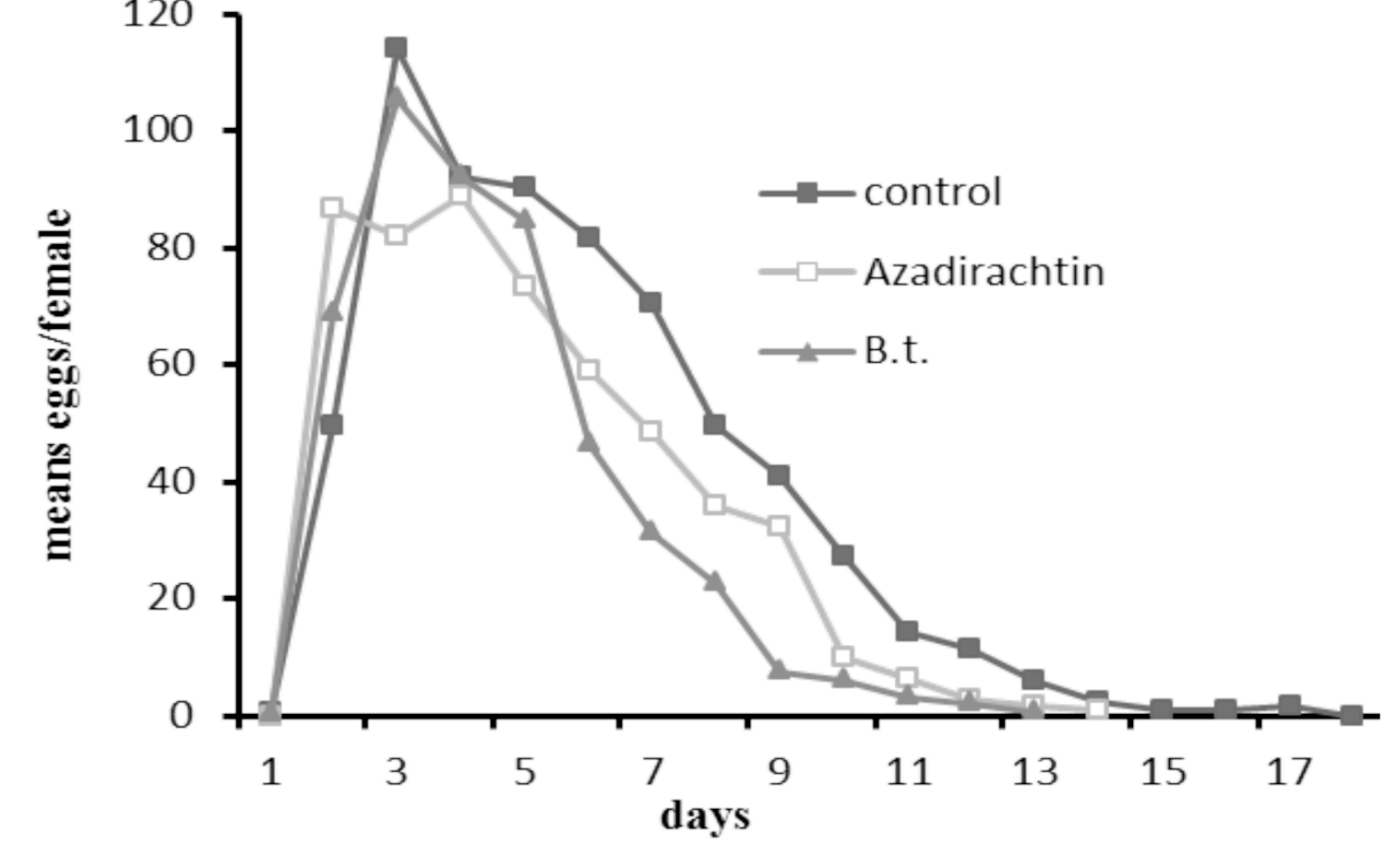
Mean eggs produced by *Helicoverpa armigera* adults that emerged from third instar larvae treated with azadirachtin and *Bt* . High quality figures are available online

The effects of azadirachtin and *Bt* on pupation and emergence rate of *H. armigera* are shown in Figure 2 . The pupation ratio was 92.4, 71.6, and 65.8% for the control, *Bt* , and azadirachtin, respectively. There was significant reduction in treatments compared with the control. The adult emergence ratio was not affected significantly by *Bt* . Higher oviposition rates were observed in the third and fourth days after adult emergence in all treatments ( Figure 3 ).

Adults of several important lepidopteran pests have been reported previously to suffer reduced fecundity after exposure to pesticides ( Pineda et al. 2009 ). In the present study also, azadirachtin and *Bt* reduced the fecundity and the pupation ratio of *H. armigera* .

### Interaction effects

The mortality percentage of third instar larvae of *H. armigera* on the seventh day after exposure to LC20 of azadirachtin, *Bt* , and a mixture of azadirachtin and *Bt* is shown in Table 4 . The interaction effects of insecticides caused 56.7% mortality on third instar larvae ( Table 4 ). Singh et al. (2007) examined combinations of lethal and sublethal concentration of azadirachtin and *Bt* subspecies *kurstaki* against first to fourth instar larvae of *H. armigera* . Their results showed that *Bt* and azadirachtin combinations of LC50 and EC20 and LC50 and EC50 caused 100% mortality. Also, the mortality was significant in LC20 and EC20 and LC20 and EC50 mixtures. Aggarwal et al. (2006) evaluated the effects of azadirachtin and *Bt* and a combination of *Bt* and azadirachtin against second and fourth instar larvae of *H. armigera* feeding on *Vicia faba* under laboratory conditions. The mortality rates caused by azadirachtin were 34% and 7% on second and fourth instar larvae, respectively, and the mortality rates caused by *Bt* were 50% and 14% on second and fourth instar larvae, respectively. The maximum mortalities of 58% and 27% on second and fourth instar larvae, respectively, were obtained in the *Bt* and azadirachtin treatment. The effects of azadirachtin products, such as neem leaf extract, neem seed kernel extract, and neem oil, were evaluated alone and in combinations at the concentrations of 5% of each treatment on second and fourth instar larvae of *H. armigera* by feeding the insect with treated chickpea ( Wakil et al. 2008 ). There was a significant difference in the mortality cased by all treatments, and the second instar larvae were more susceptible to azadirachtin products. The combinations may be useful for controlling cotton bollworm populations that have acquired resistance to *Bt* , as they may not survive the effect of the mixture.

**Table 4.**
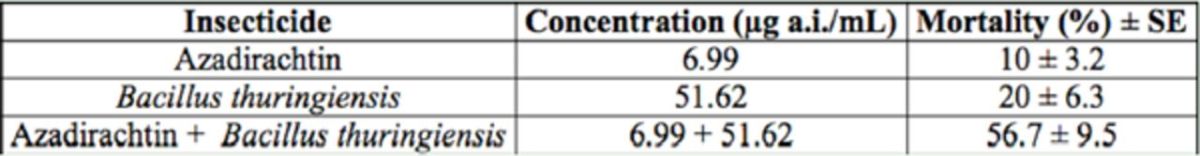
Percentage mortality ± SE of third instar larvae of *Helicoverpa armigera* on the seventh day after treatment with LC20 of azadirachtin, *Bt* , and a combination of azadirachtin and *Bt* .

### Conclusion

The results of the present study showed that both insecticides had toxic effects on *H. armigera* . The results indicated that azadirachtin and *Bt* negatively affected the larval and pupal weight, longevity, and reproductive parameters, and increased the duration of the larval and pupal period of *H. armigera* . The present study revealed that both insecticides and their combination have high potential for controlling *H. armigera* . After laboratory studies, more attention should be devoted on semi-field and field evaluations to obtain more applicable results.
